# Vaping and Health: What Do We Know about E-Cigarettes?

**DOI:** 10.1289/ehp.122-A244

**Published:** 2014-09-01

**Authors:** Carrie Arnold

**Affiliations:** Carrie Arnold is a freelance science writer living in Virginia. Her work has appeared in *Scientific American*, *Discover*, *New Scientist*, *Smithsonian*, and more.

As a pulmonologist with the San Diego Veteran’s Affairs hospital, Laura Crotty Alexander has probably answered every possible question about smoking. Whether her patients were looking for ways to quit or simply wondering whether their current health problems might be related to smoking, Crotty Alexander provided answers.

A couple of years ago, however, her patients began asking new questions: Are electronic cigarettes safer than conventional cigarettes, and should they switch? “I didn’t have the answers. As a physician and a researcher, that was very frustrating,” Crotty Alexander says.

**Figure:**
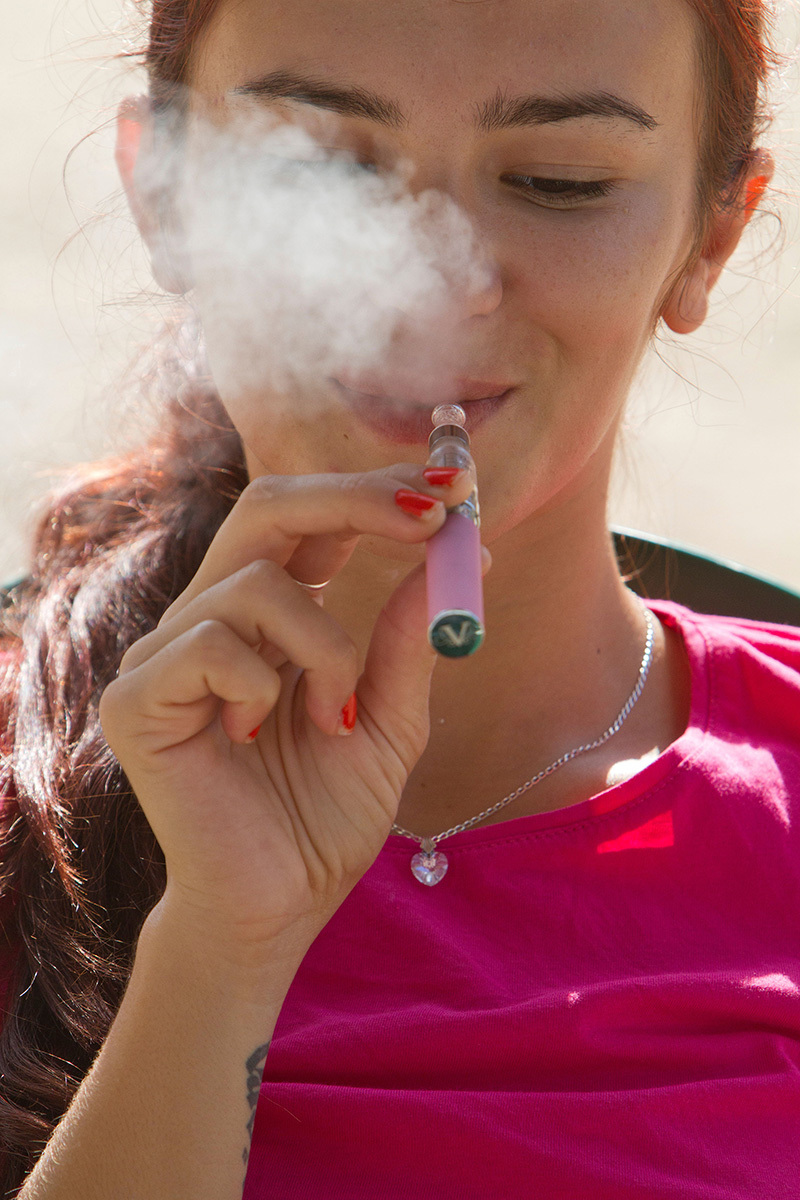
Advertisements for e-cigarettes claim they help smokers curb their habit while inhaling only “harmless water vapor,” but few tests have been conducted to confirm these claims. © Jack Ludlam/Alamy

Physicians all over the country are encountering the same questions from their patients. Out of nowhere, it seems, e-cigarettes—or electronic nicotine delivery systems, as they are formally known—are appearing at gas stations, convenience stores, and anywhere else cigarettes are sold. Marketing statements may claim e-cigarettes offer health benefits by helping smokers quit, and all e-cigarette users inhale is “harmless water vapor.”^1^

Many environmental health scientists aren’t so sure. Maciej Goniewicz, a toxicologist at the Roswell Park Cancer Institute in Buffalo, New York, says, “This is vapor, but only a small proportion of it is water.” Mostly, he says, it’s made up of propylene glycol and/or glycerin, the main ingredients in the “e-liquid” (or “e-juice”) that is vaporized inside e-cigarettes. When heated, these solvents produce an aerosol resembling cigarette smoke.^2^ Most e-liquids also contain flavorings and preservatives.^3^^,^^4^

“Most of what we know about e-cigarettes is from lab studies,” Goniewicz says. “We don’t know about the real health effects on the users of this product, especially on long-term users.”

The newness of e-cigarettes means longitudinal studies about potential health dangers are still in the distant future. Meanwhile, the existing literature about the safety of the devices consists of small studies on e-liquids and e-cigarette emissions. It remains unknown exactly how e-cigarettes and their related emissions compare with conventional cigarettes.

Despite the lack of health data, many researchers assume e-cigarettes are less dangerous than conventional cigarettes. Gerry Stimson, a public health social scientist at Imperial College London, explains, “When you burn vegetable matter, you inhale lots of nasty things into your lungs.” Because e-cigarettes only heat a liquid rather than burning tobacco leaves, he says, it creates fewer hazardous particles that can be inhaled.

“The vapor does not appear to be benign, but it does seem to be the lesser of two evils when compared to cigarettes,” Crotty Alexander says.

**Figure:**
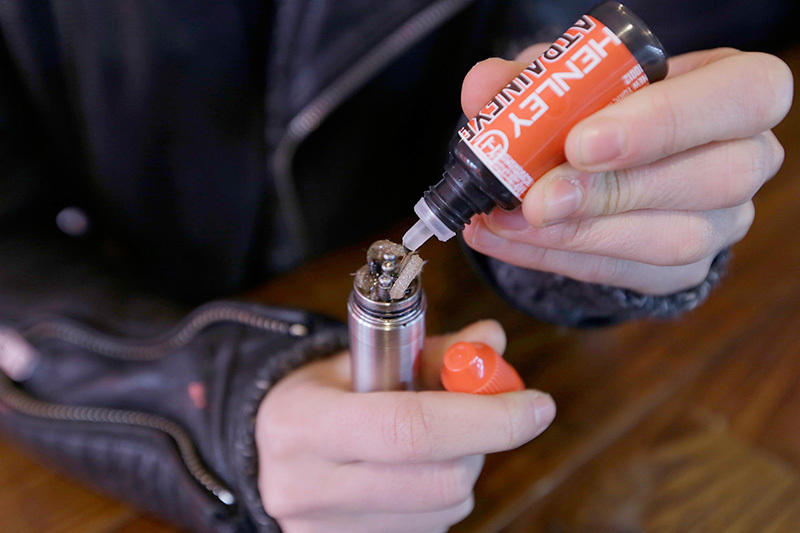
Although manufacturers offer many different designs of e-cigarettes, all involve the same basic concept: A heating element at one end aerosolizes a liquid nicotine solution, and the vapor is inhaled through a mouthpiece. © AP Photo/Frank Franklin II

Stimson adds, “At issue is a matter of weighing up potential risks against potential health benefits. Small and sometimes not so small risks are associated with all sorts of pharmacological and other health and social interventions, but the necessary precautionary principle needs to be weighed against potential benefits.”

Of course, saying something is safer than smoking cigarettes isn’t exactly setting a high bar. The Centers for Disease Control and Prevention estimates that cigarette smoking causes one in five U.S. deaths each year, including deaths resulting from secondhand smoke exposure.^5^ Smoking is a leading risk factor in chronic obstructive pulmonary disease, lung cancer, and cardiovascular disease.^6^ It’s the leading preventable cause of premature death in the United States and one of the leading causes around the world.^6^

## A Boom in Popularity

Against a backdrop of increasing awareness of the health dangers of cigarettes and legal crackdowns on public smoking, Chinese pharmacist Hon Lik first developed an electronic alternative to traditional cigarettes in 2003.^7^ E-cigarettes entered the U.S. market in 2007.^8^

The devices come in a variety of shapes and sizes, but all are variations on the same general theme: A heating element at one end aerosolizes a liquid nicotine solution, and the vapor is inhaled through a mouthpiece. “We see e-cigarettes as a single group of products, but there are hundreds of brands and many different generations and models,” Goniewicz says. “There are also huge variations in how people use these products.”

E-cigarettes were originally sold almost exclusively online and were not covered by existing tobacco regulations. At first, their popularity grew slowly, as small numbers of smokers turned to them to replace or supplement their tobacco smoking habit. As companies such as Reynolds American and Lorillard began showing interest in the devices, advertising increased, and the products moved into brick-and-mortar stores.^9^ In a short time, e-cigarettes’ unconfirmed reputation as a smoking-cessation aid and a “healthy” alternative to cigarette smoking has widely increased their popularity.^10^

Manufacturers can make the nicotine solution flavorless, but many companies add flavors, ranging from the sophisticated (mint chocolate truffle and whiskey) to the baldly juvenile (bubble gum, gummy bears, and cotton candy). A congressional report from spring 2014 accused e-cigarette manufacturers of using these flavors to appeal to youth,^11^ a marketing strategy that is prohibited for tobacco cigarettes because it is so effective at attracting young users.^12^ In contrast to tobacco products, e-cigarette sales are not age-restricted, and in 2012 an estimated 1.78 million students in grades 6–12 had tried the devices.^13^

Increases in “vaping” (as e-cigarette users call their habit) have not been matched by available knowledge about the physiological effects of the practice. And when investigators tried to quantify exposures in e-cigarette users, they rapidly ran into trouble, says tobacco researcher Stanton Glantz of the University of California, San Francisco.

For one thing, each manufacturer of e-cigarettes has a different design for the device and e-liquid,^14^ which alters how much of the vapor and its chemical load is inhaled with each puff.^15^ An individual’s unique vaping behaviors also help determine how much they inhale.^16^ The labels on refill cartridges don’t always accurately reflect the amount of nicotine found in the e-liquid,^2^^,^^17^^,^^18^^,^^19^ nor does the amount of nicotine found in the liquid appear to correlate with the amount of nicotine found in the vapor.^20^

## What We’ve Learned So Far

Although these difficulties have slowed researchers in their studies, they haven’t stopped them. Goniewicz and others started with what they already knew. Previous research on propylene glycol, one of the most commonly used constituents of e-liquids, showed it can cause eye and lung irritation.^21^ In its product safety assessment for propylene glycol, the Dow Chemical Company recommends individuals avoid inhaling the chemical.^22^

A new study by Goniewicz and colleagues in *Nicotine & Tobacco Research* reveals that potentially toxic carbonyls can form when e-liquids are heated to high temperatures. In early models of e-cigarettes, the heating element didn’t get warm enough to create these compounds. However, some newer “variable voltage” models allow users to increase the temperature of the heating element to deliver more nicotine—which also generates carbonyls.^23^

Carbonyls, which consist of a carbon atom double-bonded to an oxygen atom, are found in a variety of organic and organometallic compounds. The carbonyls identified by Goniewicz and colleagues included formaldehyde, acetaldehyde, acetone, and butanol. Propylene glycol–based e-liquids generated higher levels of carbonyls than other fluids, with levels of carcinogenic formaldehyde observed in the range seen in tobacco smoke.^23^

Interestingly, the researchers also noted that one e-liquid produced no detectable carbonyls at higher temperatures. This fluid was predominantly polyethylene glycol and contained less propylene glycol and glycerin than the other samples.^23^

Other investigators are interested in the flavorings and preservatives used in e-liquids. Although the U.S. Food and Drug Administration (FDA) classifies these additives as “generally recognized as safe,” this classification typically is based on ingestion, whereas inhalation may create a different toxicity profile.^14^ A few studies have identified various nicotine-related degradation products and other impurities in e-liquids and vapors,^17^^,^^18^^,^^24^ although some researchers have concluded these impurities occur at levels unlikely to cause harm.^3^

**Table 1:** Comparison of sample toxicants emitted by tobacco cigarettes and e-cigarettes

**Toxic compound**	**Tobacco cigarette** (μg in mainstream smoke)	**E-cigarette** (μg per 15 puffs*)	**Average ratio** (conventional vs electronic cigarette)
Formaldehyde	1.6–52	0.20–5.61	9
Acetaldehyde	52–140	0.11–1.36	450
Acrolein	2.4–62	0.07–4.19	15
Toluene	8.3–70	0.02–0.63	120
NNN**	0.005–0.19	0.00008–0.00043	380
NNK**	0.012–0.11	0.00011–0.00283	40
* The authors assumed smokers of e-cigarettes would take an average of 15 puffs per vaping session, corresponding to smoking one tobacco cigarette. ** Tobacco-specific nitrosamine, a carcinogenic compound that originates in the curing and processing of tobacco. Adapted from Goniewicz et al. (2014)^4^			

*In vitro* research has indicated the potential for cytotoxic effects of e-liquid flavorings. In one study investigators screened 35 samples of different e-cigarette solutions in three types of cells: human pulmonary fibroblasts, human embryonic stem cells, and mouse neural stem cells. Although the nicotine in these e-liquids didn’t show evidence of cytotoxicity, some of the flavorings did. Both types of stem cells were also far more sensitive to the chemicals than the adult lung cells.^25^ However, far more research is needed to confirm these findings and, if confirmed, what they mean for human health.

Fine and ultrafine particles produced during combustion of plant matter are one of the major contributors to respiratory and cardiovascular risk from smoking tobacco.^26^ Although e-cigarettes don’t involve combustion, they do still produce particles of various types.^9^ A team of researchers from Washington University in St. Louis reported that ultrafine particles of water, nicotine, and solvent appeared to deposit in the lungs in a similar pattern as the ultrafines found in tobacco smoke.^26^

In a 2013 study, cell biologist Prue Talbot of the University of California, Riverside, found another type of nanoparticle in the vapor from e-cigarettes: Analysis revealed a high concentration of heavy metals and silicates. It turned out these metal nanoparticles came from the heating element, which consisted of a nickel–chromium wire coated in silver and soldered with tin. During exposure to the heating element, the e-liquid appeared to pick up bits of metal, which then were carried in the aerosol.^27^

## Exposure Symptoms

Despite the lack of human health studies, reports from e-cigarette users indicate the potential for adverse side effects. When Talbot surveyed three different online vaping forums, she found 405 mentions of symptoms after using e-cigarettes. Although 78 were positive, and 1 was neutral, the other 326 symptoms were negative, with users most frequently complaining of headache, respiratory tract irritation, and changes in appetite.^28^

Given the popularity of e-cigarettes among teens and young adults, safety studies in adult users—even if they existed—would not necessarily reflect potential health risks of e-cigarettes for younger populations, according to allergist and pediatrician Chitra Dinakar of Children’s Mercy Hospital in Kansas City, Missouri. “Generally, young people are more sensitive to chemicals,” Dinakar says.

Kevin Chatham-Stephens, an officer with the Epidemic Intelligence Service at the Centers for Disease Control and Prevention, is tracking calls to poison control centers in relation to e-cigarette exposures. Last spring he published the first data on child exposures to e-cigarettes and their components. In the *Morbidity and Mortality Weekly Report*, Chatham-Stephens and colleagues reported that calls to U.S. Poison Control Centers related to e-cigarettes increased from 1 call in September 2010 to 215 in February 2014. Just over half the reported e-cigarette exposures reported were to the e-liquids or the vapor. He says, “We want to generate awareness for clinicians and consumers about potential health risks, and to keep in mind potential adverse health effects.”

**Figure:**
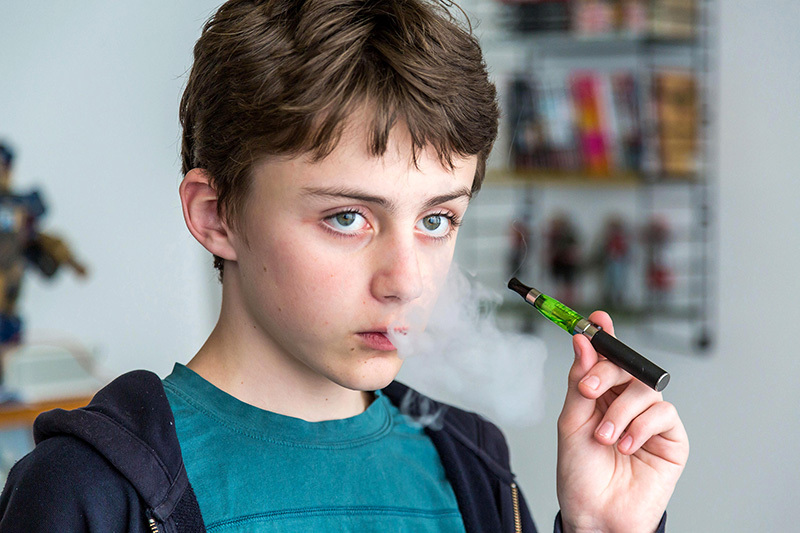
Unlike tobacco products, e-cigarettes are not age-restricted. Use among youth approximately doubled between 2011 and 2012, by which time an estimated 1.78 million students in grades 6–12 had tried the devices, according to the Centers for Disease Control and Prevention. © Phanie/Alamy

**Figure:**
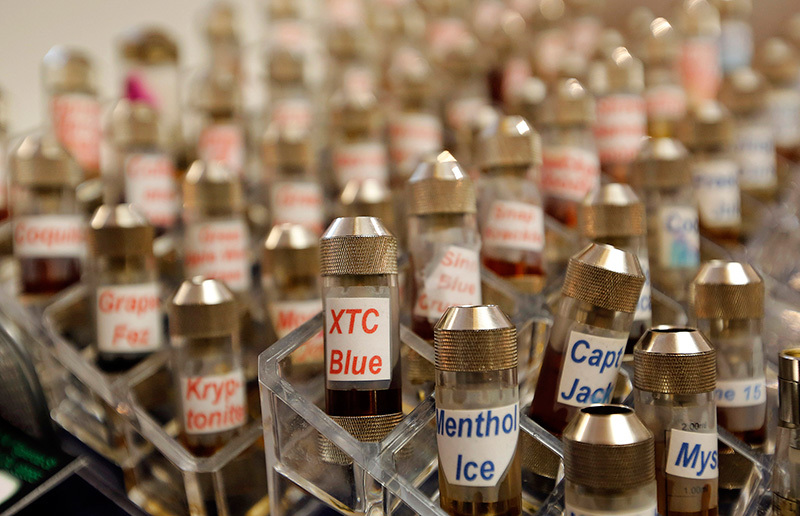
E-liquids come in hundreds of varieties, many with names and flavors that appear to target youth. Flavors besides menthol are banned from use in conventional cigarettes because they are so effective at easing children into tobacco use. © AP Photo/Reed Saxon

At this point physicians are most concerned about acute nicotine toxicity, symptoms of which can include agitation, rapid heartbeat, seizures, nausea, and vomiting.^30^ The authors of a case report of nicotine poisoning in an infant call on doctors to educate patients about the hazard posed to children by nicotine solution. They point out that nicotine solution at a strength used in some refill cartridges can be lethal if ingested (the case they reported was nonfatal).^30^

E-cigarettes may also expose bystanders to emissions, although research in this area is only just beginning. One team of researchers observed increased indoor air levels—albeit less than those associated with tobacco cigarettes—of coarse particulate matter, polycyclic aromatic hydrocarbons, and aluminum following indoor vaping sessions lasting two hours each.^31^

“E-cigarettes do appear to pollute the air, though not as much as conventional cigarettes,” Glantz says. “Many of the effects of secondhand smoke on the cardiovascular system have highly nonlinear dose–response curves,” he says, so even lower levels of e-cigarette emissions should be taken seriously. He adds, “We now have much cleaner indoor air [as a result of widespread bans on public smoking], so I can’t see why you would want to re-introduce polluted air with e-cigarettes.”

## Interim Advice

Many questions remain about whether e-cigarettes are actually safe or simply less harmful than tobacco cigarettes, and debate rages about whether or how the devices should be regulated.^32^ But the ongoing uncertainty hasn’t appeared to dampen their popularity.

Although researchers are still waiting on data about long-term health effects from e-cigarettes, Crotty Alexander has begun to provide some advice on the devices to her patients. “I don’t like to use the word ‘safe’ with e-cigarettes,” she says, “but I do tell my patients that they might be better off if they switched from regular cigarettes to e-cigarettes.”

For their part, Glantz and colleagues advise health care providers to read between the lines when a patient asks about e-cigarettes. “A patient who asks a clinician about using the e-cigarette for quitting smoking may be signaling readiness to quit smoking,” they wrote in a May 2014 clinicians’ brief.^33^ “It is most important to support the patient’s quit attempt and to try to ensure that any advice given does not undermine the patient’s motivation to quit smoking.”
