# Erratum: “Vaping and Health: What Do We Know about E-Cigarettes?”

**DOI:** 10.1289/ehp.122-a297

**Published:** 2014-11-01

The September 2014 News article “Vaping and Health: What Do We Know about E-Cigarettes?” [Environ Health Perspect 122:A244–A249 (2014); http://dx.doi.org/10.1289/ehp.122-A244] has been revised to correct errors and clarify certain statements. The article incorrectly referred twice to e-cigarette emissions as “secondhand smoke.” However, e-cigarettes do not produce smoke; they produce vapor. In addition, “Advertisements claim e-cigarettes offer health benefits by helping smokers quit” should have been attributed to reference 1, and reference 1 itself should have indicated that the cited marketing statements were provided as an example. Finally, the statement “One team of researchers observed increased levels—albeit less than those associated with tobacco cigarettes—of coarse particulate matter, polycyclic aromatic hydrocarbons, and aluminum following indoor vaping sessions lasting two hours each” should have specified that researchers observed these increased levels *in indoor air*.

*EHP* regrets the errors.

